# Serum chloride trajectory and 28-day mortality in critically ill congestive heart failure: A retrospective multicenter cohort study deriving from MIMIC-IV and externally validated

**DOI:** 10.1097/MD.0000000000049260

**Published:** 2026-06-19

**Authors:** Yu Xiang, Zhanfang Zhu, Ying Lv, Xiaoxiang Liu, Botao Li, Fuqiang Liu, Ping Yuan, Ruochen Zhang

**Affiliations:** a Department of Cardiology, Shaanxi Provincial People’s Hospital, Xi’an, China; b Department of Internal Medicine, Xi’an Jiaotong University Hospital, Xi’an, China; c Department of Nephrology, Xi’an XD Group Hospital, Xi’an, China.

**Keywords:** congestive heart failure, group-based trajectory modeling, intensive care unit, mortality, serum chloride trajectory

## Abstract

Congestive heart failure (CHF) is associated with high morbidity, mortality, and substantial economic burden. Although hypochloremia has been linked to adverse outcomes, the relationship between longitudinal serum chloride trajectories and prognosis in CHF is inadequately defined. Using the Medical Information Mart for Intensive Care IV (MIMIC-IV) database, we identified 2526 adults with a documented diagnosis of CHF who remained in the intensive care unit (ICU) for ≥6 days; an additional 226 CHF patients with ≥5 days of ICU stay were enrolled from our hospital for external validation. Group-based trajectory modeling was applied to daily serum chloride measurements obtained during the first 6/5 days of ICU admission to derive distinct trajectory classes. Survival differences across trajectories were visualized with Kaplan–Meier curves and tested by log-rank statistics. Multivariable Cox proportional-hazards models were fitted to estimate hazard ratios (HRs) for mortality across classes. Subgroup and sensitivity analyses were conducted to assess the robustness of the findings. This study included 2526 MIMIC participants (mean age 70 years, 59.1% male) and 226 validation participants (70 years, 40.3% male). Three distinct trajectories were identified in both the MIMIC and validation cohorts: Class 1 (declining chloride trajectory), Class 2 (stable chloride trajectory), and Class 3 (rising chloride trajectory). Kaplan–Meier analysis showed that Class 3 had the steepest decline in survival (log-rank *P* < .001). In fully adjusted models, Class 3 exhibited increased mortality at 28 days (MIMIC cohort, HR = 1.39, 95% confidence interval = 1.13–1.71, *P* = .002; validation cohort, HR = 1.25, 95% confidence interval = 1.05–1.49, *P* = .013), whereas Class 1 did not differ from Class 2. The absolute risk increase for Class 3 versus Class 2 was 9.65% in the MIMIC cohort and 22.21% in the validation cohort. Findings were robust across subgroups and sensitivity analyses. A rising chloride trajectory during the initial ICU stay is independently associated with higher 28-day mortality in critically ill CHF patients. These findings generate the hypothesis that careful management of progressive chloride rises may be beneficial, but this requires prospective testing in randomized trials.

## 1. Introduction

Congestive heart failure (CHF) is a complex clinical syndrome characterized by impaired ventricular filling or ejection, rendering the heart unable to meet the metabolic demands of the body. The Global Burden of Disease Study 2021 estimates that more than 55 million people worldwide now live with heart failure; this prevalence has more than doubled since 1990. South Asia has experienced the fastest growth, with an average annual increase of 0.4, driven by population aging and improved survival after acute coronary syndromes.^[1]^ Despite steady advances in guideline-directed drug and device therapies, the 5-year all-cause mortality of CHF remains 40% to 50%, higher than that of most solid cancers.^[2]^ Beyond its direct lethality, heart failure markedly increases the risks of renal failure, atrial fibrillation, stroke, and sudden cardiac death, and it accounts for roughly US $284.17 billion in annual global direct and indirect healthcare expenditures.^[3–6]^ Consequently, refining risk stratification and identifying modifiable early predictors have become central goals in heart failure research. Over the past decade, investigators have proposed biomarkers such as natriuretic peptides, soluble ST2, and high-sensitivity troponins, as well as multidimensional scoring systems like the Meta-Analysis Global Group in Chronic Heart Failure and the Seattle Heart Failure Model.^[7–10]^ Yet these metrics add only modest predictive value beyond routine variables and are constrained by cost, limited availability, or challenges to serial monitoring.

Serum chloride is the most abundant anion in extracellular fluid. Beyond maintaining acid-base and osmotic balance, it directly influences myocardial contraction and relaxation by modulating cardiomyocyte membrane potential, tubuloglomerular feedback, and sympathetic nerve activity.^[11]^ Recently, its prognostic value in critical illness has gained attention: in sepsis, admission hypochloremia predicts intensive care unit (ICU) death independently of sodium and lactate levels^[12]^; in chronic kidney disease, persistent low chloride accelerates tubulointerstitial fibrosis via activation of the renin-angiotensin-aldosterone system.^[13]^ In heart failure, a single-center retrospective study found that hypochloremia (<96 mEq/L) in ICU patients with HF was associated with higher in-hospital mortality.^[14]^ However, a single static chloride value cannot capture the dynamic electrolyte remodeling that occurs in CHF patients as a result of diuretic use, dietary changes, fluctuations in renal function, and neurohormonal rhythms. Recent methodological advances, such as group-based trajectory modeling (GBTM), now allow longitudinal biomarker trajectories to be mapped, and their predictive power is markedly superior to that of single-point measurements.^[15,16]^ Therefore, the present study will use serial serum chloride measurements to construct chloride-trajectory subtypes in patients with CHF and verify their independent predictive value for all-cause death.

This study aims to leverage the Medical Information Mart for Intensive Care IV (MIMIC-IV) database and external validation data to characterize the heterogeneity of serum chloride trajectories and evaluate the association between distinct trajectory classes and the risk of all-cause mortality.

## 2. Materials and methods

### 2.1. Data source and participant selection

This retrospective cohort study utilized the MIMIC-IV (version 2.2; MIT Laboratory for Computational Physiology and Beth Israel Deaconess Medical Center, available via PhysioNet) database, which integrates de-identified electronic health records from over 50,000 adult admissions to the Beth Israel Deaconess Medical Center between 2008 and 2019.^[17]^ Laboratory results, vital signs, medications, procedures, comorbidities, and in-hospital mortality were extracted from charted data. The MIMIC-IV database has been approved by the Massachusetts Institute of Technology Institutional Review Board and the Beth Israel Deaconess Medical Center Institutional Review Board. Informed consent was waived because all data are publicly available and de-identified in accordance with the Health Insurance Portability and Accountability Act Safe Harbor provision. We accessed MIMIC-IV via PhysioNet after completing the Collaborative Institutional Training Initiative “Data or Specimens Only Research” course (Record ID: 50081635).

For the external validation cohort, the study was approved by the Shaanxi Provincial People’s Hospital Ethics Committee (approval no: 2022XXGK008). Written informed consent was obtained from all participants or their legally authorized representatives prior to enrollment, in accordance with the Declaration of Helsinki. The studies were conducted in accordance with local legislation and institutional requirements.

**Figure 1.**
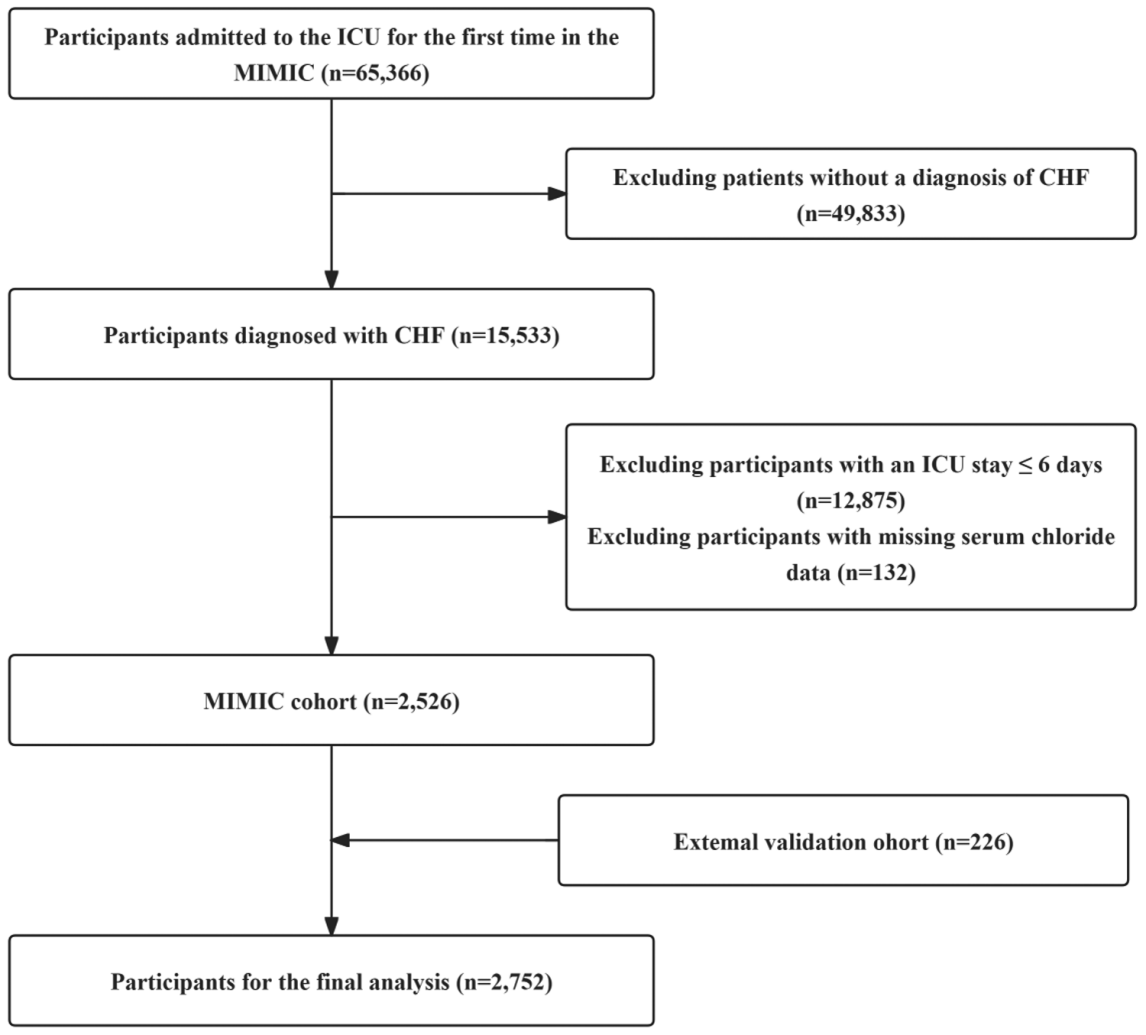
Flowchart of our participants. CHF = congestive heart failure, ICU = intensive care unit, MIMIC = Medical Information Mart for Intensive Care.

Starting with 65,366 adult patients in the MIMIC-IV database whose first ICU stay was recorded, we identified 15,533 who carried an explicit diagnosis of CHF. After excluding individuals whose ICU stay lasted ≤6 days (n = 12,875), whose serum chloride data were missing (n = 132), 2526 patients remained and formed the MIMIC cohort. In addition, we collected clinical data from patients diagnosed with CHF at Shaanxi Provincial People’s Hospital between July 2022 and June 2025. The primary outcome was 28-day all-cause mortality, ascertained via telephone follow-up. Individuals lacking serum chloride values within 5 days before ICU admission or whose 28-day vital status could not be determined were excluded, leaving 226 participants for the analysis (validation cohort). A total of 2752 CHF patients were ultimately included (Fig. 1).

### 2.2. Data collection

We used PostgreSQL (version 14.2; PostgreSQL Global Development Group ) to query the MIMIC-IV relational database. The following variables were extracted for the first 24 hours after ICU admission: demographics-age, sex, weight; vital signs-heart rate, systolic blood pressure, diastolic blood pressure, respiratory rate; laboratory results-hemoglobin, white blood cells (WBC), platelets, anion gap, blood urea nitrogen (BUN), creatinine, glucose, sodium, potassium, calcium; and severity scores-Sequential Organ Failure Assessment, and Simplified Acute Physiology Score II. Comorbidities (renal disease, cerebrovascular disease, chronic pulmonary disease, diabetes, hypertension, ischemic stroke, malignant cancer) were retrieved from the same source. Therapeutic measures include fluid balance, continuous renal replacement therapy, and diuretics.

We extracted the following baseline variables within 24 hours of ICU admission from the Shaanxi Provincial People’s Hospital database for CHF patients: age, sex, weight, heart rate, respiratory rate, WBC, sodium, fluid balance, diabetes, hypertension, continuous renal replacement therapy, diuretics, and 28-day mortality.

Variables with a missing rate exceeding 20% were excluded outright; for those with <20% missing values, multiple imputation was performed using the random-forest method.

### 2.3. Exposure and outcome

The exposure of the study was the longitudinal trajectory of serum chloride. Daily serum chloride values were extracted for each patient during the first 6 days (MIMIC cohort) or 5 days (validation cohort) following ICU admission. In the MIMIC cohort, chloride was measured at the discretion of the treating clinician (1–2 measurements per day); for days with multiple measurements, the daily mean was used. In the validation cohort, serum chloride was routinely measured every morning during the ICU stay. GBTM was used to identify trajectory classes. Models specifying 1 to 5 classes were fitted; the final model was selected on the basis of the lowest Bayesian information criterion while ensuring that each trajectory class comprised ≥5% of the cohort, the threshold was applied for below methodological reasons. First, classes representing <5% of the sample are statistically unstable, with wide confidence intervals (CIs) for parameter estimates, compromising the reliability of subsequent regression analyses. Second, from a clinical standpoint, a trajectory class affecting fewer than 5% of patients has limited generalizability and practical relevance for risk stratification in critically ill CHF populations.^[15,16,18]^ The primary outcomes were all-cause mortality at 28 days after ICU admission.

### 2.4. Statistical analysis

Continuous variables across different chloride trajectories were compared with one-way analysis of variance or Kruskal–Wallis tests (normally distributed data are presented as mean ± standard deviation, non-normally distributed data as median [interquartile range]); categorical variables are shown as n (%) and were compared with χ^2^ tests.

Kaplan–Meier curves for 28 survival were plotted, and differences among trajectories were assessed with the log-rank test. Using Class 2 (stable chloride trajectory) as a reference, Cox proportional-hazards models were constructed: Model 1 was unadjusted; Model 2 was adjusted for age, sex, and weight; and Model 3 additionally included comorbidities and laboratory parameters. Furthermore, we calculated absolute risk differences and numbers needed to harm for 28-day mortality across trajectory classes.

Robustness was examined through subgroup and sensitivity analyses in the MIMIC cohort.

All analyses were performed in R (version 4.2.0; R Foundation for Statistical Computing); two-sided *P* < .05 was considered statistically significant.

## 3. Results

### 3.1. Three serum chloride trajectories

**Figure 2.**
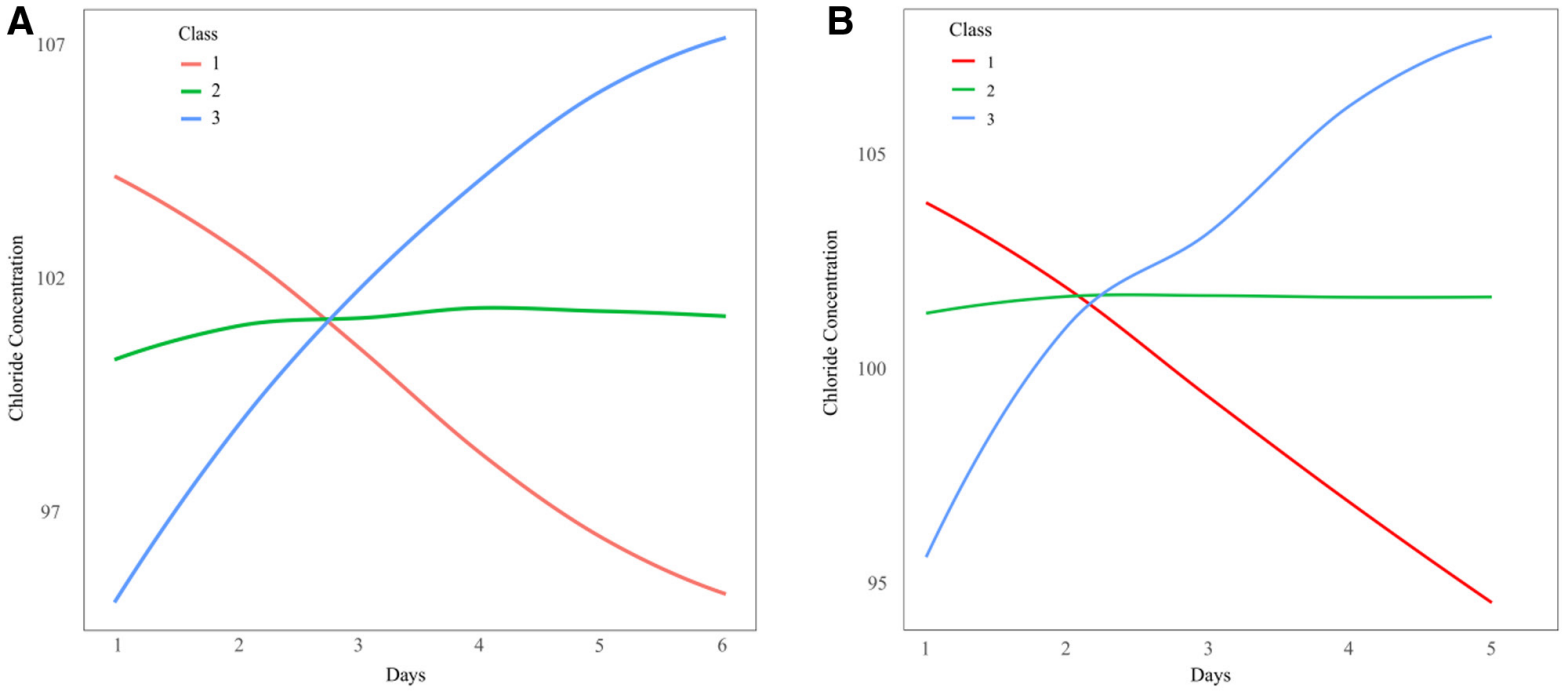
Serum chloride trajectories identified by GBTM in critically ill CHF patients (A: MIMIC cohort, B: validation cohort). CHF = congestive heart failure, GBTM = group-based trajectory modeling, MIMIC = Medical Information Mart for Intensive Care.

GBTM identified 3 optimal serum chloride trajectories in critically ill CHF patients in both the MIMIC and validation cohorts (Fig. 2 and Table 1): Class 1 (declining chloride trajectory) exhibited a sustained fall in serum chloride; Class 2 (stable chloride trajectory) maintained chloride levels with minimal fluctuation; and Class 3 (rising chloride trajectory) showed a progressive increase, with all posterior probabilities > .80 (Table 2).

**Table 1:** Selection of trajectory models for serum chloride in patients with CHF.

Number of groups	BIC	Log-likelihood	Class 1	Class 2	Class 3	Class 4	Class 5
MIMIC cohort							
1	89,798.26	−44,883.46	100.00	–	–	–	–
2	87,170.23	−43,557.69	58.12	41.88	–	–	–
3	86,317.61	−43,119.63	24.28	60.53	15.20	–	–
4	85,971.90	−42,935.03	9.27	50.83	35.75	4.15	–
5	85,828.16	−42,851.40	4.12	45.13	34.01	15.12	1.62
Validation cohort							
1	6623.00	−3300.66	100	–	–	–	–
2	6478.06	−3220.06	71.24	28.76	–	–	–
3	6413.43	−3179.61	19.03	68.58	12.39	–	–
4	6420.12	−3174.83	45.13	12.39	33.19	9.29	–
5	6435.59	−3174.43	8.85	14.60	48.23	19.47	8.85

BIC = Bayesian information criterion, CHF = congestive heart failure, MIMIC = Medical Information Mart for Intensive Care.

**Table 2:** Posterior probabilities in the 3-trajectory model.

	Prob 1	Prob 2	Prob 3
MIMIC cohort			
Class 1	0.87	0.13	0.00
Class 2	0.07	0.89	0.04
Class 3	0.00	0.11	0.89
Validation cohort			
Class 1	0.87	0.13	0.00
Class 2	0.06	0.90	0.04
Class 3	0.00	0.09	0.91

MIMIC = Medical Information Mart for Intensive Care.

### 3.2. Baseline characteristics

Table 3 presents the baseline characteristics of the 2526 critically ill CHF patients stratified by the 3 GBTM-identified serum chloride trajectories in the MIMIC cohort. The average follow-up duration was 23.79 days. Compared with Classes 2 and 1, Class 3 had significantly higher WBC, anion gap, BUN, and creatinine (all *P* < .05). Sequential Organ Failure Assessment scores were highest in class 3 (*P* < .05). Comorbidity analysis showed renal disease, cerebrovascular disease, and ischemic stroke were more frequent in Class 3 than in the other 2 groups. Moreover, patients in Class 3 exhibited the lowest proportion of diuretic use. Follow-up outcomes revealed 28-day mortality rates of 35.94% in Class 3, the highest among the 3 trajectories, whereas Class 1 had the lowest rates, with the differences being statistically significant (*P* < .001).

**Table 3:** Baseline characteristics of critically ill CHF patients grouped by serum chloride trajectories in MIMIC cohort.

	Total (n = 2526)	Class 1 (n = 613)	Class 2 (n = 1529)	Class 3 (n = 384)	*P*
Age (yr)	70.56 ± 13.72	70.36 ± 14.11	70.92 ± 13.52	69.44 ± 13.86	.156
Sex, n (%)					.218
Male	1492 (59.07)	345 (56.28)	911 (59.58)	236 (61.46)	
Female	1034 (40.93)	268 (43.72)	618 (40.42)	148 (38.54)	
Weight (kg)	87.02 ± 27.27	88.14 ± 29.67	86.50 ± 26.49	87.29 ± 26.33	.446
Heart rate (min)	72.20 ± 16.05	72.71 ± 15.39	71.85 ± 16.17	72.77 ± 16.59	.399
SBP (mm Hg)	85.45 ± 16.50	84.35 ± 15.43	85.78 ± 16.86	85.91 ± 16.63	.160
DBP (mm Hg)	43.80 ± 10.99	43.39 ± 10.58	44.13 ± 11.16	43.14 ± 10.93	.163
Respiratory rate (min)	12.73 ± 4.06	12.46 ± 3.98	12.86 ± 4.10	12.66 ± 4.04	.117
Hemoglobin (g/dL)	9.88 ± 2.35	9.69 ± 2.32	10.00 ± 2.33	9.70 ± 2.43	.007
WBC (K/µL)	11.53 ± 7.61	11.53 ± 6.26	11.25 ± 7.32	12.65 ± 10.21	.005
Platelet (K/µL)	183.10 ± 98.60	177.53 ± 98.60	185.33 ± 97.05	183.12 ± 104.45	.254
Anion gap (mmol/L)	13.43 ± 3.87	12.55 ± 3.72	13.44 ± 3.65	14.77 ± 4.55	<.001
BUN (mg/dL)	33.51 ± 24.08	30.70 ± 22.11	32.98 ± 23.71	40.14 ± 27.26	<.001
Creatinine (mg/dL)	1.72 ± 1.58	1.55 ± 1.43	1.68 ± 1.50	2.16 ± 2.01	<.001
Glucose (mg/dL)	126.24 ± 45.10	124.83 ± 40.79	126.83 ± 46.72	126.18 ± 45.17	.651
Sodium (mmol/L)	136.44 ± 5.47	138.51 ± 4.79	136.53 ± 4.87	132.82 ± 6.77	<.001
Potassium (mmol/L)	3.97 ± 0.61	4.02 ± 0.62	3.96 ± 0.60	3.95 ± 0.64	.083
Calcium (mg/dL)	8.09 ± 0.89	8.07 ± 0.84	8.10 ± 0.91	8.06 ± 0.88	.751
Fluid balance (mL)	2364.52 ± 4581.01	2772.96 ± 3780.40	2232.00 ± 4627.95	2196.71 ± 5537.00	.051
SOFA	6.98 ± 3.67	7.23 ± 3.45	6.74 ± 3.70	7.55 ± 3.82	<.001
SAPS II	44.97 ± 14.55	44.74 ± 13.96	44.74 ± 14.75	46.28 ± 14.65	.159
Renal disease, n (%)					.024
No	1535 (60.77)	386 (62.97)	939 (61.41)	210 (54.69)	
Yes	991 (39.23)	227 (37.03)	590 (38.59)	174 (45.31)	
Cerebrovascular disease, n (%)					.006
No	2018 (79.89)	507 (82.71)	1225 (80.12)	286 (74.48)	
Yes	508 (20.11)	106 (17.29)	304 (19.88)	98 (25.52)	
Chronic pulmonary disease, n (%)					.607
No	1574 (62.31)	388 (63.30)	941 (61.54)	245 (63.80)	
Yes	952 (37.69)	225 (36.70)	588 (38.46)	139 (36.20)	
Diabetes, n (%)					.529
No	1476 (58.43)	369 (60.20)	889 (58.14)	218 (56.77)	
Yes	1050 (41.57)	244 (39.80)	640 (41.86)	166 (43.23)	
Hypertension, n (%)					.356
No	1936 (76.64)	473 (77.16)	1159 (75.80)	304 (79.17)	
Yes	590 (23.36)	140 (22.84)	370 (24.20)	80 (20.83)	
Ischemic stroke, n (%)					.002
No	2303 (91.17)	577 (94.13)	1389 (90.84)	337 (87.76)	
Yes	223 (8.83)	36 (5.87)	140 (9.16)	47 (12.24)	
Malignant cancer, n (%)					.392
No	2309 (91.41)	568 (92.66)	1389 (90.84)	352 (91.67)	
Yes	217 (8.59)	45 (7.34)	140 (9.16)	32 (8.33)	
Diuretics, n (%)					.012
No	1341 (53.09)	299 (48.78)	818 (53.50)	224 (58.33)	
Yes	1185 (46.91)	314 (51.22)	711 (46.50)	160 (41.67)	
CRRT, n (%)					.131
No	2031 (80.40)	480 (78.30)	1249 (81.69)	302 (78.65)	
Yes	495 (19.60)	133 (21.70)	280 (18.31)	82 (21.35)	
28-d mortality, n (%)					<.001
No	1833 (72.57)	460 (75.04)	1127 (73.71)	246 (64.06)	
Yes	693 (27.43)	153 (24.96)	402 (26.29)	138 (35.94)	

BUN = blood urea nitrogen, CRRT = continuous renal replacement therapy, DBP = diastolic blood pressure, MIMIC = Medical Information Mart for Intensive Care, SAPS II = Simplified Acute Physiology Score II, SBP = systolic blood pressure, SOFA = Sequential Organ Failure Assessment, WBC = white blood cell count.

Table 4 presents the baseline characteristics of the 226 critically ill CHF patients stratified by the 3 GBTM-identified serum chloride trajectories in the validation cohort. The average follow-up duration was 23.57 days. Compared with Classes 2 and 1, Class 3 had significantly higher sodium and 28-day mortality (*P* < .05).

**Table 4:** Baseline characteristics of critically ill CHF patients grouped by serum chloride trajectories in validation cohort.

	Total (n = 226)	Class 1 (n = 43)	Class 2 (n = 155)	Class 3 (n = 28)	*P*
Age (yr)	70.82 ± 13.53	69.62 ± 13.50	71.47 ± 13.16	69.07 ± 15.73	.560
Sex, n (%)					.598
Male	91 (40.27)	19 (44.19)	59 (38.06)	13 (46.43)	
Female	135 (59.73)	24 (55.81)	96 (61.94)	15 (53.57)	
Weight (kg)	87.68 ± 26.55	88.48 ± 23.03	88.32 ± 28.01	83.03 ± 23.24	.625
Heart rate (min)	91.12 ± 20.99	88.56 ± 22.46	91.47 ± 19.83	93.14 ± 25.06	.626
Respiratory rate (min)	20.39 ± 6.39	19.02 ± 6.48	20.56 ± 6.39	21.54 ± 6.14	.226
WBC (K/µL)	12.61 ± 8.68	11.94 ± 7.42	12.43 ± 5.90	14.60 ± 18.38	.409
Sodium (mmol/L)	138.54 ± 5.65	140.77 ± 5.87	138.45 ± 5.23	135.64 ± 6.29	<.001
Fluid balance (mL)	2372.64 ± 3186.14	2874.45 ± 3180.13	2349.70 ± 3140.21	1568.70 ± 3472.85	.312
Diabetes, n (%)					.533
No	124 (54.87)	24 (55.81)	82 (52.90)	18 (64.29)	
Yes	102 (45.13)	19 (44.19)	73 (47.10)	10 (35.71)	
Hypertension, n (%)					.621
No	77 (34.07)	13 (30.23)	56 (36.13)	8 (28.57)	
Yes	149 (65.93)	30 (69.77)	99 (63.87)	20 (71.43)	
Diuretics, n (%)					.094
No	127 (56.19)	18 (41.86)	91 (58.71)	18 (64.29)	
Yes	99 (43.81)	25 (58.14)	64 (41.29)	10 (35.71)	
CRRT, n (%)					.289
No	185 (81.86)	35 (81.40)	130 (83.87)	20 (71.43)	
Yes	41 (18.14)	8 (18.60)	25 (16.13)	8 (28.57)	
28-d mortality, n (%)					.038
No	70 (75.22)	31 (72.09)	123 (79.35)	16 (57.14)	
Yes	56 (24.78)	12 (27.91)	32 (20.65)	12 (42.86)	

CHF = congestive heart failure, CRRT = continuous renal replacement therapy, WBC = white blood cell count.

### 3.3. Serum chloride trajectories and outcome

**Figure 3.**
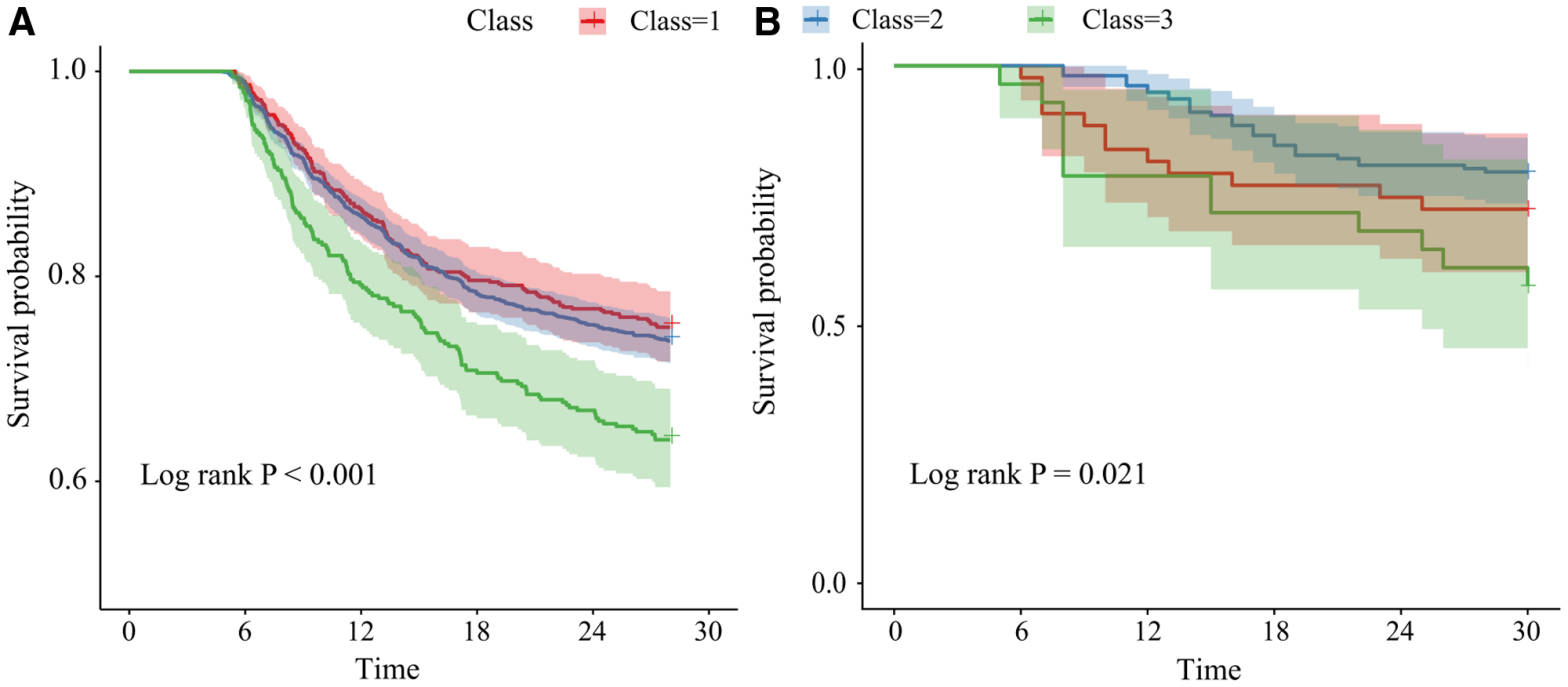
Kaplan–Meier analysis results of different serum chloride trajectories and mortality in critically ill CHF patients (A: MIMIC cohort, B: validation cohort). CHF = congestive heart failure, MIMIC = Medical Information Mart for Intensive Care.

In Figure 3, Kaplan–Meier curves showed that the 3 serum chloride trajectories were significantly associated with 28-day mortality risk (log-rank *P* < .05). In both the MIMIC and validation cohorts, survival probability fell most rapidly in Class 3, whereas the decline was gentler in Classes 1 and 2.

Table 5 summarizes the results of 3 multivariable Cox regression models examining the association between serum chloride trajectories and mortality risk in critically ill CHF patients. After full adjustment for demographics, comorbidities, and all laboratory parameters (Model 3), compared with Class 2 (stable chloride trajectory), Class 3 (rising chloride trajectory) showed a significantly higher 28-day mortality risk (MIMIC cohort, hazard ratio [HR] = 1.39, 95% CI = 1.13–1.71, *P* = .002; validation cohort, HR = 1.25, 95% CI = 1.05–1.49, *P* = .013). In contrast, Class 1 (declining chloride trajectory) did not differ significantly from Class 2 in 28-day mortality risk. A consistent trend was observed in minimally adjusted Model 1 and in Model 2 adjusted for age, sex, and body weight, with Class 3 consistently showing the highest mortality risk (all *P* < .05). To facilitate bedside clinical interpretation, we computed absolute risk differences and numbers needed to harm alongside HRs. In the MIMIC cohort, compared with Class 2, Class 3 was associated with an absolute risk increase of 9.65 percentage points (95% CI = 4.38%–14.72%), corresponding to a number needed to harm of 10.4 (95% CI = 6.8–22.8). In other words, for every 10 patients classified into the rising chloride trajectory rather than the stable chloride trajectory, approximately 1 additional death would be expected within 28 days. In the validation cohort, the absolute risk increase for Class 3 versus Class 2 was even more pronounced at 22.21 percentage points (95% CI = 2.75%–41.67%), with a number needed to harm of 4.5 (95% CI = 2.4–36.4), indicating that approximately 1 additional death would occur for every 4 to 5 patients in the rising chloride trajectory. Class 1 showed no significant difference from Class 2 in both cohorts.

**Table 5:** Relationship between serum chloride trajectories and 28-mortality in patients with CHF in different models.

Class	Model 1		Model 2		Model 3		ARD (95% CI)	NNH (95% CI)
	HR (95% CI)	*P*	HR (95% CI)	*P*	HR (95% CI)	*P*		
MIMIC cohort								
2	Ref		Ref		Ref			
1	0.94 (0.78–1.13)	.516	0.95 (0.79–1.15)	.599	0.99 (0.82–1.21)	.955	−1.33% (−5.41 to 2.75)	–
3	1.46 (1.21–1.78)	<.001	1.55 (1.28–1.88)	<.001	1.39 (1.13–1.71)	.002	9.65% (4.38 to 14.72)	10.4 (6.8–22.8)
Validation cohort								
2	Ref		Ref		Ref			
1	0.91 (0.77–1.06)	.226	0.92 (0.78–1.07)	.281	1.01 (0.86–1.19)	.926	7.26% (−8.12 to 22.64)	–
3	1.40 (1.18–1.65)	<.001	1.50 (1.26–1.77)	<.001	1.25 (1.05–1.49)	.013	22.21% (2.75 to 41.67)	4.5 (2.4–36.4)

Model 1: crude.

Model 2: adjust for age, sex, and weight.

Model 3: adjust for age, sex, weight, heart rate, SBP, DBP, respiratory rate, hemoglobin, WBC, platelet, anion gap, BUN, creatinine, glucose, sodium, potassium, calcium, SOFA, SAPS II, fluid balance, renal disease, cerebrovascular, chronic pulmonary disease, diabetes, hypertension, ischemic stroke, malignant cancer, diuretics, and CRRT (MIMIC cohort); adjust for age, sex, weight, heart rate, diabetes, hypertension (validation cohort).

ARD = absolute risk difference, CHF = congestive heart failure, CI = confidence interval, HR = hazard ratio, MMIC = Medical Information Mart for Intensive Care, NNH = number needed to harm.

### 3.4. Subgroup analysis

**Figure 4.**
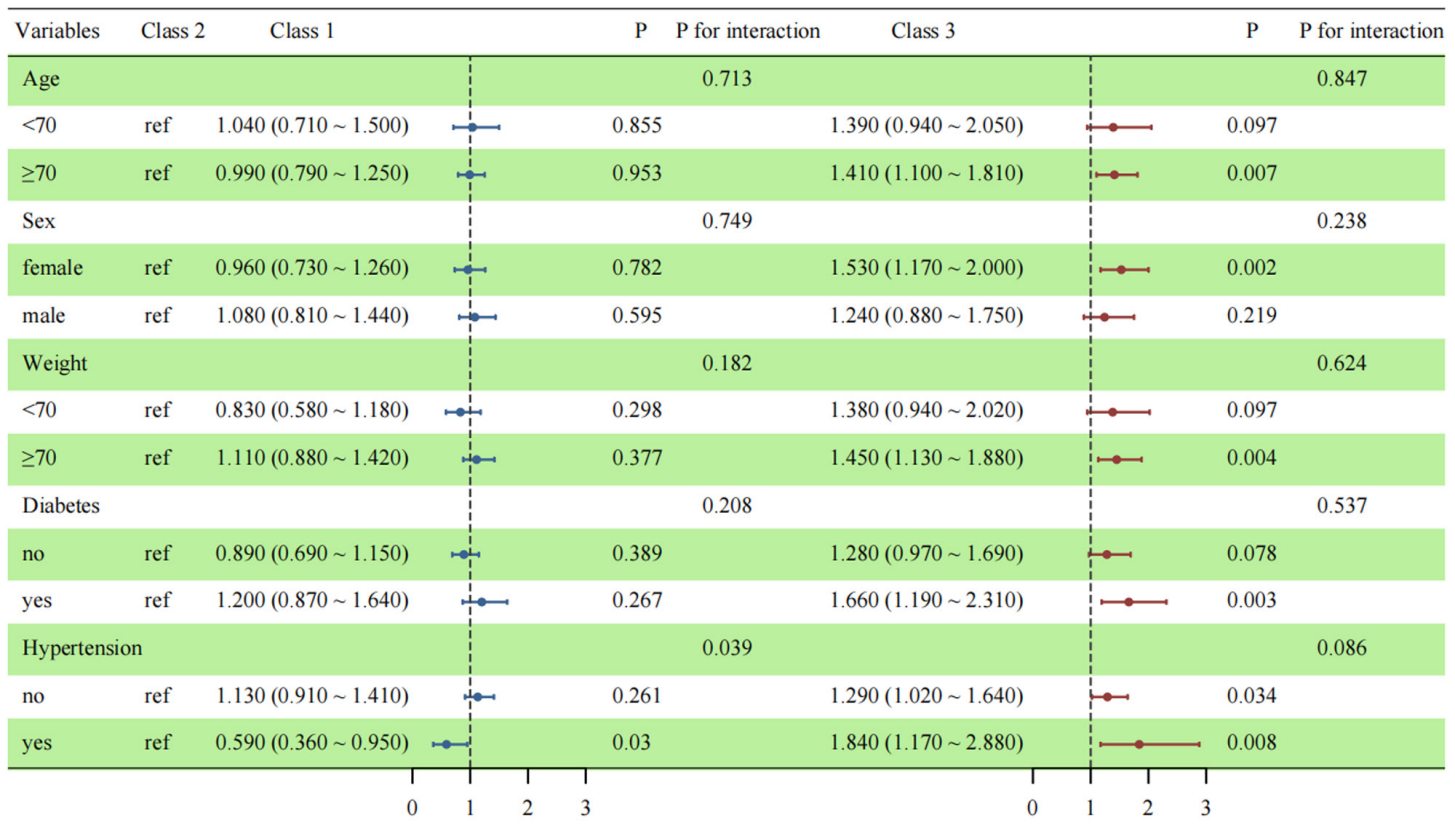
Subgroup analysis of the association between serum chloride trajectories and 28-day mortality in the MIMIC cohort. MIMIC = Medical Information Mart for Intensive Care.

Subgroup analysis showed that the association between serum chloride trajectories and mortality remained consistent across most strata at 28 days in the MIMIC cohort (Fig. 4). Regardless of age, sex, weight, or presence of diabetes, Class 3 carried a trend toward higher risk compared with Class 2. Significant interaction was observed among hypertensive patients: patients classified as Class 1 hypertension exhibited a markedly reduced 28-day mortality risk (HR = 0.59; 95% CI = 0.36–0.95; interaction *P* = .039). No significant interaction effects were observed in other subgroups.

### 3.5. Sensitive analysis

Finally, sensitivity analysis were conducted to confirm the robustness of our findings in MIMIC cohort (Table 6). After excluding patients who died within 7 days of ICU admission, Class 3 remained associated with a higher mortality risk at 28 days (HR = 1.36, 95% CI = 1.09–1.71, *P* = .007) compared to Class 2, while no significant difference was observed between Class 1 and Class 2. Similarly, after excluding patients with malignant tumors, Class 3 continued to show elevated mortality risk at 28 days (HR = 1.43, 95% CI = 1.15–1.78, *P* = .001) relative to Class 2, with no statistically significant difference between Class 1 and Class 2.

**Table 6:** Sensitivity analysis in MIMIC cohort.

	Model 1		Model 2		Model 3	
	HR (95% CI)	*P*	HR (95% CI)	*P*	HR (95% CI)	*P*
Excluding died within 7 d						
Class						
2	Ref		Ref		Ref	
1	0.96 (0.79–1.18)	.709	0.97 (0.80–1.19)	.796	1.00 (0.81–1.23)	.988
3	1.40 (1.13–1.74)	.002	1.49 (1.20–1.85)	<.001	1.36 (1.09–1.71)	.007
Excluding malignancy						
Class						
2	Ref		Ref		Ref	
1	0.89 (0.73–1.09)	.270	0.91 (0.74–1.11)	.362	0.95 (0.77–1.17)	.608
3	1.52 (1.24–1.86)	<.001	1.66 (1.35–2.04)	<.001	1.43 (1.15–1.78)	.001

Model 1: crude.

Model 2: adjust for age, sex, and weight.

Model 3: adjust for age, sex, weight, heart rate, SBP, DBP, respiratory rate, hemoglobin, WBC, platelet, anion gap, BUN, creatinine, glucose, sodium, potassium, calcium, SOFA, SAPS II, fluid balance, renal disease, cerebrovascular, chronic pulmonary disease, diabetes, hypertension, ischemic stroke, diuretics, and CRRT.

CI = confidence interval, HR = hazard ratio, MMIC = Medical Information Mart for Intensive Care.

## 4. Discussion

To the best of our knowledge, this is the first large-scale retrospective cohort study to use trajectory modeling to characterize how dynamic changes in serum chloride relate to short- and medium-term mortality in patients with CHF. We found that individuals who followed a “rising-chloride” trajectory had significantly higher all-cause mortality at 28 days, whereas no statistically significant difference was observed between the “declining-chloride” and “stable-chloride” trajectories. Moreover, the external dataset confirmed the robustness of our findings. Our findings suggest that longitudinal chloride trajectories could serve as a novel, inexpensive biomarker for early risk stratification and personalized intervention. Prior single-point studies have associated admission hypochloremia with in-hospital mortality in acute heart failure, a pattern that aligns with our findings. We now show that CHF patients who present with low serum chloride on ICU admission and experience a rise over the next 6 days face increased 28-day mortality. These results complement earlier static reports and highlight the need to monitor patients with low chloride after ICU admission. While preventing progressive chloride rises appears intuitively appealing as a potential means of reducing short-term death, this hypothesis requires rigorous testing in randomized controlled trials before clinical implementation.^[19,20]^

In recent years, trajectory analysis has been increasingly applied in cardiovascular research to uncover how dynamic exposures – such as blood pressure, lipids, and inflammation-shape clinical endpoints. Wu et al modeled 5 trajectories of cardiovascular health score among 74,701 Chinese adults and found that maintaining a high cardiovascular health score was associated with a significantly lower incidence of cardiovascular disease between 2010 and 2015.^[21]^ Another Chinese cohort showed that elderly individuals whose systolic blood pressure followed a “Excess BP with decreasing trend” pattern experienced a 34% higher all-cause mortality and a 67% higher cardiovascular mortality after accounting for competing risks.^[22]^ In the lipid field, a Guangzhou Medical University team used the ELSA database to construct plasma atherogenic index trajectories and reported a 33% higher cardiovascular event rate in the “high-stable” group versus the “low-stable” group.^[23]^ Regarding inflammation, veno-arterial extracorporeal membrane oxygenation patients whose interleukin-6 trajectory was “ascending” had roughly double the 28-day mortality of those with the “stable” or “descending” patterns.^[24]^ Collectively, these studies indicate that trajectories capture cumulative cardiometabolic risk better than single measurements, yet longitudinal investigations of electrolytes remain scarce. Our study is the first to apply GBTM to critically ill CHF patients, mapping early ICU-day chloride dynamics and demonstrating that a “rising-chloride trajectory” independently predicts 28-day mortality, thereby furnishing time-sensitive evidence for electrolyte management and extending the frontier of trajectory analytics in cardiovascular medicine.

Although the present study did not directly dissect the biological circuitry linking elevated chloride trajectories to excess mortality, we propose 3 mutually reinforcing explanatory pathways. First, patients within the rising chloride trajectory exhibited concomitant elevations in WBC, anion gap, BUN, and creatinine, collectively indicating a heightened systemic inflammatory burden and more advanced renal injury. Second, at the molecular level, chloride is a central determinant of volume and acid-base homeostasis; sustained increases in its plasma concentration may potentiate sodium-chloride cotransporter activity along the distal nephron, thereby impairing glomerular filtration, promoting sodium retention, and accelerating renal functional decline.^[25]^ Finally, we documented a significantly higher prevalence of coexisting renal and cerebrovascular disease among individuals with rising chloride trajectories, and these complications also increase mortality risk in CHF patients.^[26,27]^

Subgroup analyses revealed that the rising chloride trajectory was significantly associated with increased mortality among hypertensive individuals, implying that hypertension may modulate heart failure prognosis by disturbing electrolyte homeostasis. As a dominant risk factor for heart failure, chronic hypertension induces vascular and myocardial remodeling that alters renal handling of sodium and chloride, ultimately precipitating hyperchloremia.^[28]^ Concurrently, heightened sympathetic drive and activation of the renin-angiotensin system in hypertensive patients can upregulate the sodium-chloride cotransporter, exacerbating fluid retention and cardiac preload.^[29]^

The association between rising chloride trajectories and increased mortality may be partially explained by iatrogenic chloride loading from intravenous fluid therapy. Chloride-rich crystalloid solutions, such as 0.9% saline, contain supraphysiological chloride concentrations (154 mmol/L) compared with human plasma (100–110 mmol/L).^[30]^ Large-scale randomized trials and meta-analyses have demonstrated that resuscitation with chloride-liberal fluids is associated with increased risks of acute kidney injury, hyperchloremic metabolic acidosis, and mortality compared with balanced crystalloids.^[31,32]^ We observed that an upward trend in chloride ion concentration was associated with elevated WBC, anion gap, BUN, and creatinine levels. This finding aligns with the aforementioned observations, suggesting that chloride ion overload may exacerbate systemic inflammatory responses and renal dysfunction in this high-risk population.

This study has several limitations. First, the MIMIC-IV database and external validation data are retrospective, so residual confounding cannot be fully eliminated. Second, chloride measurements were ordered at the discretion of clinicians; missing values at certain time points may introduce bias. Third, the primary endpoint was all-cause mortality; future prospective studies focused on death specifically attributable to heart failure are warranted. Fourth, our inclusion criterion of ICU stay ≥6 days excluded 82.9% of CHF patients in the MIMIC-IV database, creating a substantial selection bias. Critically ill CHF patients with shorter ICU stays were excluded. Consequently, our findings primarily apply to a highly selected population of prolonged-ICU CHF patients. Fifth, we were unable to account for specific treatment variables that may influence chloride trajectories, including the type of intravenous fluids administered (e.g., 0.9% saline vs balanced crystalloids), diuretic dosing regimens, and vasopressor use. These unmeasured confounders could contribute to observed chloride rises. Future studies should incorporate granular treatment data to disentangle pathophysiological from iatrogenic chloride changes. Sixth, due to the limitations of the retrospective study, this research did not stratify patients according to heart failure subtypes (heart failure with reduced ejection fraction vs heart failure with preserved ejection fraction); however, the mechanisms underlying chloride ion metabolism levels differ among patients with different subtypes of CHF. Future studies should evaluate whether chloride trajectories carry differential prognostic value in heart failure with reduced ejection fraction versus heart failure with preserved ejection fraction. Clinicians should therefore exercise caution when extrapolating our results to shorter-stay patients.

## 5. Conclusion

In critically ill CHF patients, a rising ICU chloride trajectory independently predicted higher 28-day mortality. This large, multicenter, longitudinal study positions dynamic chloride surveillance as an inexpensive, potentially actionable biomarker for early risk stratification. However, the therapeutic utility of chloride-directed interventions remains hypothetical and must be established through prospective clinical trials.

## Acknowledgments

We thank the Shaanxi Provincial People’s Hospital for providing the external validation dataset. We also appreciate the patients and their families for their participation.

## Author contributions

**Methodology:** Yu Xiang, Ruochen Zhang.

**Project administration:** Yu Xiang, Zhanfang Zhu, Xiaoxiang Liu, Ruochen Zhang.

**Resources:** Yu Xiang, Zhanfang Zhu, Botao Li, Fuqiang Liu.

**Software:** Yu Xiang, Xiaoxiang Liu.

**Validation:** Yu Xiang, Zhanfang Zhu, Botao Li, Ruochen Zhang.

**Writing – original draft:** Yu Xiang, Zhanfang Zhu.

**Conceptualization:** Zhanfang Zhu, Ping Yuan.

**Data curation:** Zhanfang Zhu, Ping Yuan.

**Formal analysis:** Zhanfang Zhu, Ying Lv, Xiaoxiang Liu, Botao Li, Ping Yuan.

**Investigation:** Zhanfang Zhu, Ruochen Zhang.

**Supervision:** Ying Lv, Ruochen Zhang.

**Visualization:** Ying Lv, Ruochen Zhang.

**Writing – review & editing:** Ying Lv, Xiaoxiang Liu, Botao Li, Fuqiang Liu, Ping Yuan, Ruochen Zhang.

**Funding acquisition:** Fuqiang Liu, Ruochen Zhang.
